# KLF5 downregulation desensitizes castration-resistant prostate cancer cells to docetaxel by increasing BECN1 expression and inducing cell autophagy

**DOI:** 10.7150/thno.33282

**Published:** 2019-07-28

**Authors:** Jing Jia, Hai-Bao Zhang, Qi Shi, Chao Yang, Jian-Bin Ma, Bin Jin, Xinyang Wang, Dalin He, Peng Guo

**Affiliations:** 1 Department of Urology, The First Affiliated Hospital of Xi'an Jiaotong University, Xi'an, Shaanxi, China, 710061; 2 Department of Plastic, Cosmetic and Maxillofacial Surgery, The First Affiliated Hospital of Xi'an Jiaotong University, Xi'an, Shaanxi, China, 710061; 3 Key Laboratory for Tumor Precision Medicine of Shaanxi Province, Xi'an, Shaanxi, China, 710061; 4 Oncology Research Lab, Key Laboratory of Environment and Genes Related to Diseases, Ministry of Education, Xi'an, Shaanxi, China, 710061

**Keywords:** KLF5, prostate cancer, autophagy, BECN1, docetaxel

## Abstract

KLF5 is frequently deleted or downregulated in prostate cancer. However, it is not known whether downregulation of KLF5 is associated with the response of prostate cancer cells to chemotherapy and/or prognosis of patients.

**Methods:** We monitored cell growth by MTT and colony formation assays, and cell autophagy through tandem fluorescence microscopy and transmission electron microscopy. Gene expression was analyzed by RT-qPCR and Western blotting. We determined the binding of KLF5 together with HDAC3 on the beclin-1 (*BECN1*) promoter by the ChIP assay, oligonucleotides pulldown, and co-immunoprecipitation. The effect of docetaxel on cell growth *in vivo* was examined in a CWR22RV1 xenograft tumor mouse model.

**Results**: In the present study, we found that KLF5 down-regulation was associated with progression of prostate cancer and poor prognosis of patients. KLF5 knockdown reduced the sensitivity of prostate cancer cells to docetaxel *in vitro* and *in vivo*, and docetaxel treatment decreased the expression of KLF5. Moreover, we confirmed that docetaxel treatment inhibited cell death by inducing autophagy in prostate cancer cells. Thus, we hypothesized that KLF5 could be a regulator of cell autophagy. Interestingly, KLF5 could inhibit prostate cancer cell autophagy by suppressing the transcription of BECN1 cooperatively with HDAC3. Another significant finding was that docetaxel treatment repressed KLF5 expression through AMPK/mTOR/p70S6K signaling pathway resulting in increased BECN1, induction of cell autophagy, and promotion of cell survival in castration-resistant prostate cancer cells.

**Conclusions:** Our results indicated that downregulation of KLF5 promoted cell autophagy in prostate cancer. Furthermore, reduced KLF5 also facilitated cell autophagy induced by docetaxel resulting in desensitization to the drug and cell survival. Decreased levels of KLF5 led to increased BECN1 via AMPK/mTOR/p70S6K signaling. Thus, repression of BECN1 and cell autophagy was critical for KLF5 to increase the sensitivity of prostate cancer cells to docetaxel.

## Introduction

Prostate cancer is the most common tumor in males worldwide. It was estimated that about 26,730 men would die from prostate cancer in 2017 in the United States, leading to the third most frequent cause of male cancer deaths 1. Androgen deprivation therapy (ADT) is the first line therapy for most first time diagnosed prostate cancer patients. Even though more than 90% of patients initially respond to ADT, the disease progresses to castration-resistant prostate cancer (CRPC) 2. So far, only a few drugs are approved for the treatment of CRPC, such as docetaxel, which provides a significant advantage in CRPC patient survival 3. However, distinguishing between the tumors sensitive to docetaxel versus those that are insensitive remains problematic.

Autophagy or “self-eating” is an essential and highly conserved process, which maintains cellular homeostasis by degrading cytoplasmic protein waste through the lysosomal system 4, 5. In cancer, autophagy is complex and multifaceted. Reports have shown that autophagy suppresses tumor development; however, in established tumors, autophagy can meet the heightened nutrient demand of proliferative cancer cells, and help cancer cells survive during therapeutic interventions, such as radiotherapy, chemotherapy, and hormone therapy 6-8. In prostate cancer, it has been reported that suppression of autophagy can increase cell sensitivity to docetaxel treatment 9. Thus, autophagy could be an important factor in determining the sensitivity of prostate cancer cells to docetaxel 10.

KLF5 transcription factor, expressed in epithelial cells of various human organs, such as breast, colon, lung, bladder, and prostate, is involved in cell proliferation and carcinogenesis 11. In prostate cancer, the *KLF5* gene is frequently deleted and downregulated. It has been reported that chromosome 13q.21 region, which contains the *KLF5* gene, is deleted in about 39% human prostate cancers as detected by comparative genomic hybridization analysis 12. Consistently, *KLF5* gene is deleted in 10% prostate cancers according to the gene copy number assay in the TCGA (The Cancer Genome Atlas, http: //cancergenome.nih.gov/) database. Moreover, KLF5 protein is degraded by the WWP1 E3-ligase-mediated proteasome pathway in prostate cancer cells 13. Deletion of *Klf5* in the prostates of knockout mice has been reported to promote tumorigenesis initiated by *Pten* deletion 14. Therefore, KLF5 may have a tumor suppressor function in prostate cancer. However, whether the downregulation of KLF5 relates to the response of prostate cancer cells to chemotherapy and prognosis of patients is still unknown.

In the present study, we analyzed the correlation between KLF5 expression and prostate cancer prognosis and examined whether KLF5 downregulation increased cell sensitivity to docetaxel in prostate cancer cells *in vitro* and *in vivo*. Furthermore, we investigated the possible regulation of cell autophagy by KLF5 and whether its downregulation increased cell autophagy and decreased cell sensitivity to docetaxel via inducing the expression of beclin1 (BECN1), a key regulator of autophagy. Finally, we further explored whether docetaxel regulated cell autophagy and expression of KLF5 through AMPK/mTOR/p70S6K signaling. Our results suggest that the deletion and downregulation of KLF5 in prostate cancer lead to decreased response to docetaxel and poorer prognosis.

## Methods

### Cell culture

The human prostate cancer cell line, CWR22Rv1, was purchased from American Type Culture Collection (Manassas, VA, USA). C4-2B cell line was a gift from Dr. Jer-Tsong Hsieh of the University of Southwestern Medical Center. Both cell lines were cultured in RPMI1640 medium supplemented with 10% fetal bovine serum (Gibco, NY, USA) at 37°C, in humidified air containing 5% of CO_2_.

### Reagents, antibodies, and plasmids

3-methyladenine (3-MA, 189490) and Bafilomycin A1 (BAFA1, 196000) were purchased from EMD Millipore (Darmstadt, Germany). Docetaxel and PF4708671 were from Selleck Chemicals (Houston, TX, USA). Lipofectamine 2000 reagent was purchased from Invitrogen (Thermo Fisher Scientific, Inc., Waltham, MA, USA). RIPA buffer was acquired from Cell Signaling Technology (Danvers, MA, USA), protease inhibitor and phosphatase inhibitor were from Abcam (Cambridge, MA, USA). Roche (Mannheim, Germany). Primary antibodies against LC-3 I/II, Beclin1, ATG3, ATG5, ATG7, mTOR, p-mTOR (Ser2448), ACC, p-ACC (Ser79), p-AMPK(Thr172), and AMPK were acquired from Cell Signaling Technology and HDAC3 was from Abcam (Cambridge, MA, USA). PVDF membrane was purchased from Bio-Rad Laboratories, Inc. (Hercules, CA, USA). The siKLF5 #1, siKLF5 #2 siRNAs, siBeclin1 #1, siBeclin1 #2 siRNAs, siBcl2 #1, siBcl2 #2 siRNAs and the scrambled RNA, which used as shcontrol were obtained from RiboBio (Guangzhou, China). PLKO.1 lentiviral vectors encoding short hairpin RNA (shRNA) targeting non-specific control (NC) or human KLF5 (sh710: 5′-GGTTACCTTACAGTATCAACA-3′) were constructed by GenPharma (Shanghai, China).

### Lentivirus preparation and infection

PAX2, VSV-G, and the plasmids described above were co-transfected into 293T cells using the Lipofectamine 2000 reagent (Invitrogen) according to the manufacturer's protocol. After 72 hours, the supernatants were harvested and used to infect C4-2 and CW22RV1 cells in the presence of 8 μg/mL polybrene. The stable KLF5 knockdown cell clones were selected and maintained in 2-3 μg/mL puromycin.

### Bioinformatics

Prostate cancer microarray data from GEO Data Sets (GSE16560) were downloaded from the GEO website (http://www.ncbi.nlm.nih.gov/geo/) and RNA sequencing data of prostate cancer samples was downloaded from TCGA Data portal (https://tcga-data.nci.nih.gov/tcga/tcgaHome2.jsp). The data were normalized to Z score before statistical analysis.

### MTT assay

To test the cell viability, 3-(4, 5-dimethylthiazol-2-yl)-2, 5-diphenyltetrazolium bromide (MTT) assay was used. 4000 cells in 400 μl of medium per well were seeded in 96-well plates. Cells were subjected to various treatments and cultured for the indicated times, followed by incubation with 0.5 mg/mL of MTT at 37°C for 4h. The medium was replaced by 150μL DMSO per well to dissolve the precipitates. Colorimetric analysis using a 96-well micro-plate Autoreader (Bio-Tek Instruments Inc., Winooski, VT, USA) was performed at wavelength 490 nm. Independent experiments were repeated in triplicate.

### Colony formation assay

Cells transfected with indicated shRNA and sh-control (a scrambled RNA) were seeded in 6-well plate (1,000 cells/well) in 2 mL culture medium overnight. In the drug treatment group, the medium was changed with fresh medium containing docetaxel (2 nM) or vehicle (DMSO) every 2 days. All groups were allowed to grow for two weeks. Colonies were fixed with 4% paraformaldehyde and stained with crystal violet for 10 min at room temperature. Colonies consisting of more than 50 cells were counted.

### Tandem fluorescence microscopy

Cells were seeded onto coverslips and were treated with Docetaxel or DMSO for 24 h. Also, cells with KLF5 knockdown by lentivirus carrying shKLF5 and the control cells were seeded onto coverslips for 24 h. Subsequently, cells were transiently transfected with a tandem fluorescent ptfLC-3-expressing plasmid with Lipofectamine 2000 reagent for an additional 24 h. Cells were further fixed by 4% paraformaldehyde. The localization of LC-3 puncta was observed by fluorescence microscopy (×200, Olympus).

### Transmission electron microscopy (TEM)

Cells were transfected with siKLF5 #1 and a scrambled RNA control for 72 h and then fixed in Karnovsky's fixative (2% paraformaldehyde and 5% glutaraldehyde in 0.1 M cacodylate, pH 7.4) followed by osmium tetroxide. Samples were then dehydrated in ethanol, infiltrated, and embedded with TAAB low viscosity resin (TLV) mixture at 60°C for 24 h and sectioned to 80 nm in thickness on 300 mesh copper slot grids. The analysis was performed by TEM (×8,000 and ×40,000, JEOL, JEM-1400).

### Western blot analysis

Cells were treated with designated treatments and then washed with ice-cold phosphate-buffered saline and then solubilized in RIPA buffer containing protease and phosphatase inhibitors. Next, proteins were loaded onto 10% SDS-PAGE and transferred to PVDF membranes, which were immunoblotted with indicated primary antibodies (dilution: 1:1,000) overnight at 4°C followed by peroxidase-conjugated secondary antibody (dilution: 1:3,000) for 1 h at room temperature. The bands were visualized by the ECL system (Bio-Rad).

### Reverse transcription-quantitative real-time polymerase chain reaction (RT-qPCR) analysis

RT‑qPCR was performed using a previously described method 15. Total RNA from cells was extracted using fasten 2000 RNA extract kit following the manufacturer's protocol. Reverse transcription was performed with 2 μg RNA using Takara reverse kit (Takara Biotechnology Co., Ltd., Dalian, China). SYBR green reaction mix (Takara) was used to perform RT-PCR following the manufacturer's instructions. Primer sequences used are shown in Table S1. 18S was used as an internal control.

### Dual luciferase activity assay

*BECN1* promoter report plasmid pGL3-V9955-2 was generated by inserting a 948 bp of its promoter region into the pGL3-basic plasmid. To perform promoter luciferase assay, pGL3-control, pGL3-basic or pGL3-V1.7 were co-transfected with HDAC3 into KLF5-knockdown subclones of C4-2 and CW22RV1 cells or KLF5-overexpressing 293T cells using X-tremeGENE HP DNA transfection reagent (Roche, Mannheim, Germany). Luciferase assay was carried out using the Dual Luciferase Assay kit (Promega, Madison, WI, USA) following the manufacturer's instructions. Three wells of cells were used for each data point.

### Chromatin immunoprecipitation (ChIP) assay

ChIP assay was performed in normal cultured C4-2 and CW22RV1 cells using SimpleChIP® Enzymatic Chromatin IP Kit (Magnetic Beads) from Cell Signaling Technology following the manufacturer's protocol. Antibody against KLF5 or HDAC3 and normal rabbit IgG were used to precipitate protein/DNA complex. Precipitated DNA was analyzed by PCR with region-specific primers (Table S2).

### Oligonucleotides pulldown assay

Oligonucleotides for the *BECN1* promoter (-255 to +132), with biotin-labeled on the 5'-end of primers (the specific sequence showed in Table S3), were synthesized by GENEWIZ (Suzhou, China). KLF5 was knocked down in prostate cancer cells before cells were lysed. Procedures for pull-down DNA-bound proteins were detailed in our previous study 16. Finally, the KLF5 protein and HDAC3 protein were detected on the same membrane by Western blot analysis.

### Co-immunoprecipitation

Cells were harvested and lysed using cell lysis buffer (50 mM Tris-HCl, pH 7.5, 150 mM NaCl, 1% Nonidet P-40, 0.5% sodium deoxycholate, and 1% protease inhibitor cocktail, Sigma-Aldrich). Cell lysates were centrifuged, and the supernatants were incubated with indicated antibodies and Protein G Plus beads (Calbiochem) at 4°C overnight. The beads were washed three times with cell lysis buffer, and the precipitated proteins were further analyzed. For Western blotting, equal amounts of protein (80-100 micrograms) from cell lysates were denatured in sample buffer (Thermo Fisher Scientific) and subjected to SDS-polyacrylamide gel electrophoresis. The Flag-linked KLF5 and pcDNA3-linked HDAC3 were further detected.

### Xenograft tumor model

Animal experiments were performed according to procedures approved by the Institutional Animal Care and Use Committee of Xi'an Jiaotong University. For tumorigenesis assay in nude mice, 2×10^6^ cells were injected subcutaneously into one side of the flank region. Ten mice were used for each cell clone. Docetaxel was dissolved in DMSO and administered intraperitoneally to mice at the concentration of 15 mg/kg body weight, once a week, for 4 weeks started from 1 week after cells injection. DMSO alone was used as the control. Xenograft tumors were harvested, weighed, and fixed with 4% paraformaldehyde after 5 weeks.

### Immunohistochemistry

Tumor sections of nude mice xenografts were analyzed by immunohistochemistry (IHC) using EnVision^TM^ System (DAKO, Carpinteria, CA, USA). Primary antibodies used in IHC were KLF5 (Abcam, 1:200), Beclin-1 (CST, 1:200) and ATG5 (CST, 1:200). Immunohistochemistry was performed following the previously described method 16.

### Statistical analysis

GraphPad Prism version 6.0 software (GraphPad, San Diego, CA, USA) was used to analyze differences between two groups (Student's t-test) and Pearson's correlation and linear regression analyses were performed. A p value less than 0.05 was considered to be significant.

## Results

### Low KLF5 expression correlates with poor prognosis of prostate cancer

**Figure 1:**
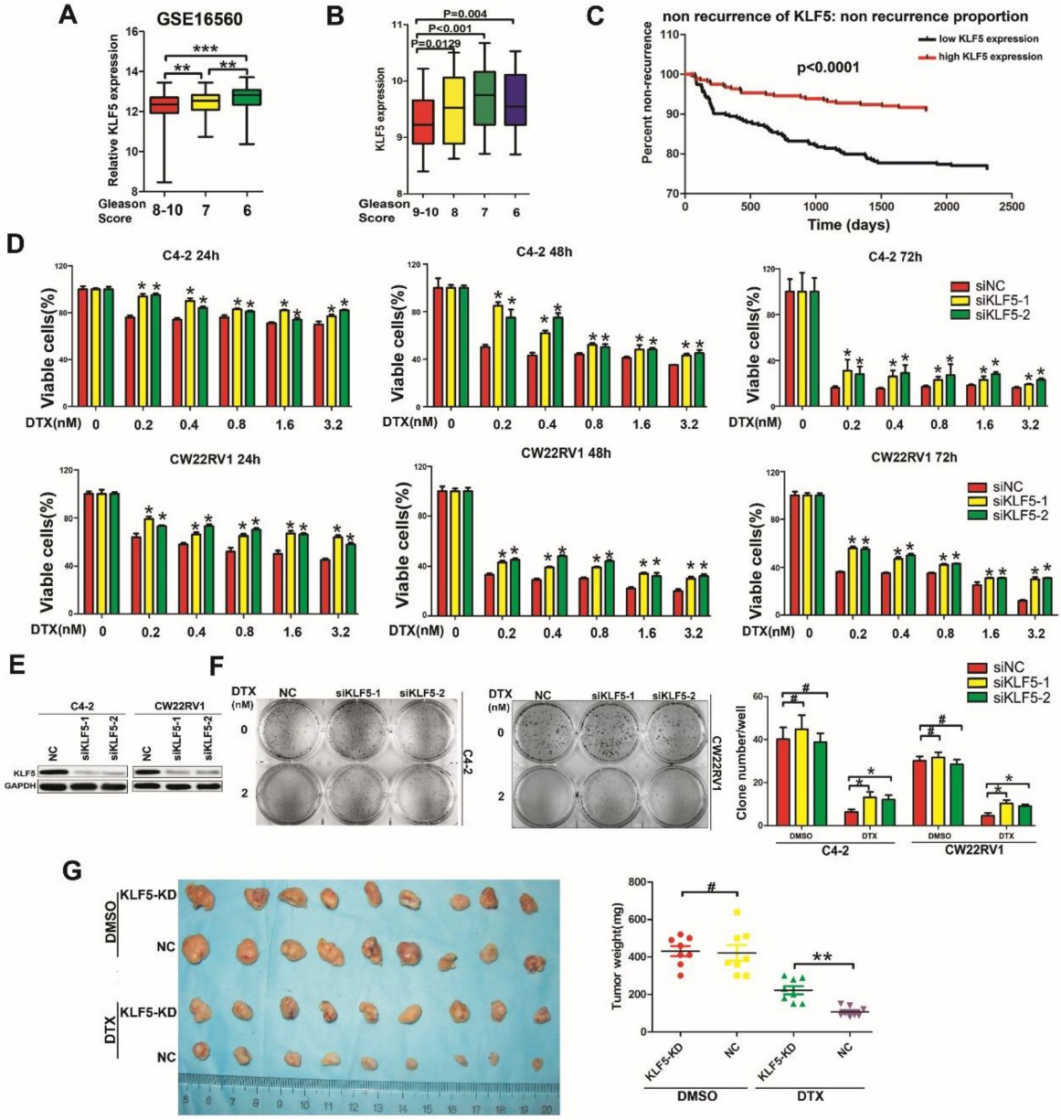
** Low KLF5 expression is associated with poor prognosis of prostate cancer and sensitivity to docetaxel in prostate cancer cells. (A)** Relative expression of KLF5 in patients with different Gleason Scores. Gleason Score 6:n=83; Gleason Score 7:n=117; Gleason Score 8-10:n=81. Data from GEO profiles. **(B)** Relative expression of KLF5 in patients with different Gleason Scores. Gleason Score 6:n=39; Gleason Score 7:n=194; Gleason Score 8:n=40; Gleason Score 9-10:n=87. Data from the TCGA database. **(C)** Kaplan-Meier analysis of the time to first recurrence of prostate cancer patients with high KLF5 expression (n=275) and low KLF5 expression (n=274) levels: the *p*-value is depicted. Data from the TCGA database. **(D and E)** Cells transfected with siKLF5 or a scrambled RNA for 24 h and then treated with various concentrations of docetaxel for different times. Cell viability was assessed by the MTT assay (D) and knockdown of KLF5 was validated by Western blotting (E). **(F)** In colony formation assay, cells were transfected with siKLF5 or a scrambled RNA and then treated with docetaxel (2 nM) for two weeks, colonies were stained with crystal violet and statistically analyzed. **(G)** Mice injected with CW22RV1/shNC (NC) and CW22RV1/shKLF5 (KLF5-KD) cells were treated with docetaxel (15 mg/kg body weight) or DMSO for 4 weeks and subsequently, the subcutaneous xenografts were harvested and weighted. The values are shown as the mean ± SD. #*p*>0.05, * p<0.05, ** p<0.01.

According to the GEO database, low expression of KLF5 was associated with high-grade tumors (Figure 1A, GSE16560, http://www.ncbi.nlm.nih.gov/geo/). To confirm this result, we also examined microarray data profiling of human prostate cancer tissues from the TCGA database to ascertain the correlation between KLF5 expression and Gleason Score in prostate cancer. (https://cancergenome.nih.gov/). As shown in Figure 1B, low KLF5 expression was associated with high Gleason Score of prostate cancer. Further analysis showed that patients with higher KLF5 expression had longer times to the first recurrence compared with patients with lower KLF5 expression (Figure 1C, TCGA data, 549 patients in total, https://cancergenome.nih.gov/). Thus, clinical evidence supports that KLF5 might play a role in prostate cancer progression and low KLF5 expression is associated with poor prognosis.

### Down-regulation of KLF5 leads to resistance to docetaxel in prostate cancer cells

Docetaxel is a second line therapy for prostate cancer patients who are not suitable for androgen deprivation therapy, such as CRPC patients. C4-2 and CWR22RV1 prostate cancer cells express androgen receptor, but their growth does not depend on androgen, making them appropriate cell models to study CRPC. To explore whether KLF5 is involved in cell sensitivity to docetaxel in prostate cancer cells, we knocked down KLF5 and treated cells with docetaxel at different concentrations. KLF5 knockdown resulted in lower cell sensitivity to docetaxel in C4-2 and CWR22RV1 prostate cancer cells as detected by cell viability assay (Figure 1D-1E). To further detect the reduction of cell sensitivity by KLF5 down-regulation in the long-term, we performed colony formation assay and found that KLF5 knockdown significantly promoted colony formation when C4-2 and CWR22RV1 prostate cancer cells were treated with docetaxel for two weeks. Interestingly, KLF5 knockdown did not cause significantly less colony formation compared with the negative control cells (Figure 1F).

Furthermore, we examined whether KLF5 knockdown decreased cell sensitivity to docetaxel in the CW22RV1 cell xenograft model by treating the nude mice with 15 mg/kg body weight of docetaxel for 4 weeks. We found the tumor weights in the CW22RV1/shKLF5 (KLF5-KD) group (222.5 ± 61.82 mg, n = 9) to be dramatically higher than those in CW22RV1/shNC (NC) group (107.125 ± 26.36 mg, n = 9) (Fig. 1G). On the other hand, KLF5 knockdown did not change tumor weights between the CW22RV1/shKLF5 (KLF5-KD) group: 431.25 ± 75.68 mg, n = 9 and CW22RV1/shNC (NC) group: 421.25 ± 118.74 mg, n = 9 in nude mice treated with DMSO (Figure 1G). These results indicated that KLF5 knockdown also decreased prostate cancer cell sensitivity to docetaxel *in vivo*. In summary, our results demonstrated that KLF5 knockdown decreases the sensitivity of prostate cancer cells to docetaxel both *in vitro* and *in vivo*.

### Docetaxel downregulates KLF5 expression and induces cell autophagy

**Figure 2:**
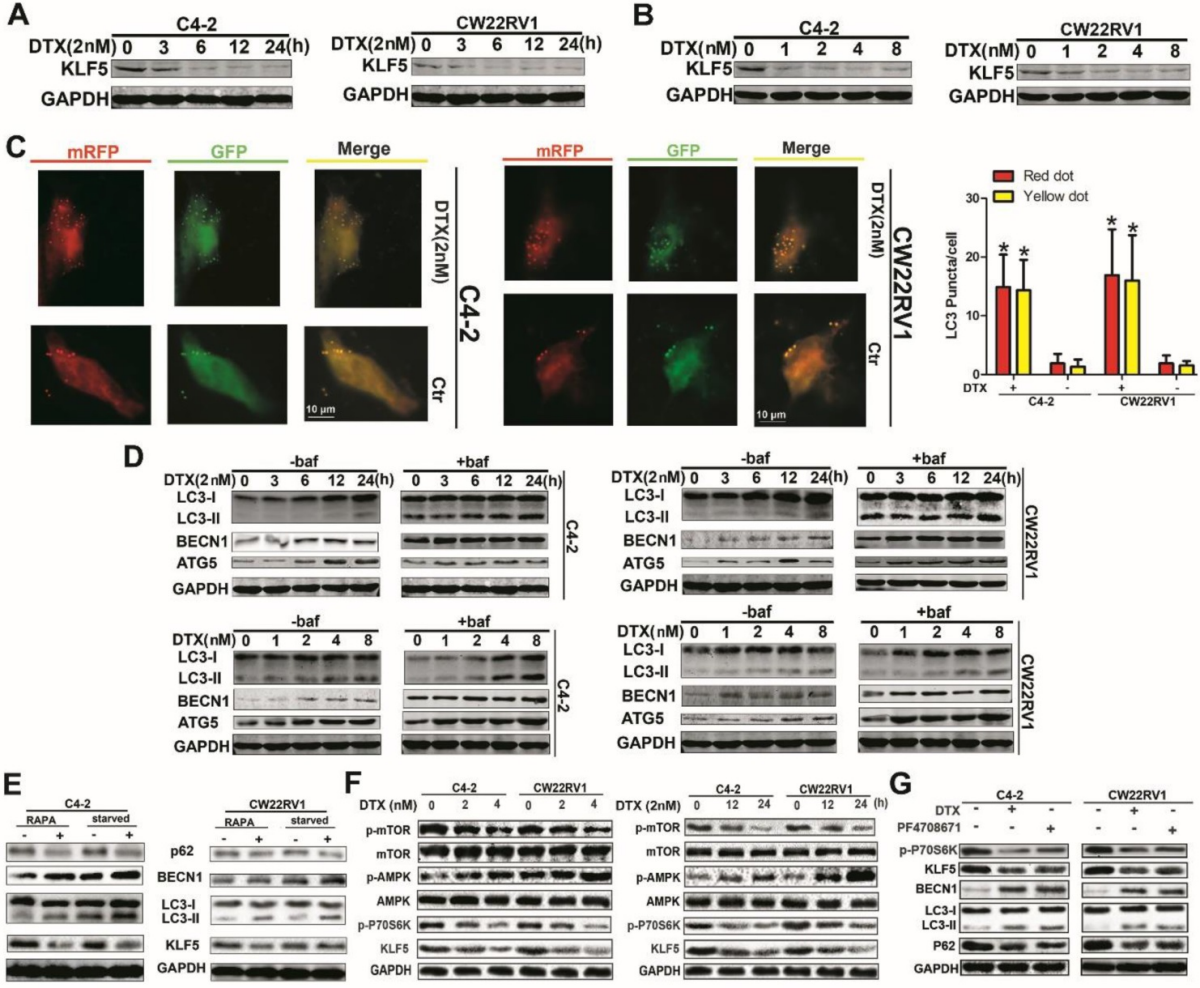
** Docetaxel reduces expression of KLF5 and increases cell autophagy in C4-2 and CW22RV1 cells. (A and B)** Cells treated with various concentrations of docetaxel for 4 8h or treated with docetaxel (2 nM) for different times. KLF5 protein level was detected by Western blotting. **(C)** Examples of cells treated with docetaxel for 24 h and then transiently transfected with ptfLC-3 plasmid for another 24 h by fluorescence microscopy (×400). Yellow dots point to autophagosomes while red dots point to autolysosomes. Right: Quantification of the number of autophagosomes (yellow LC-3 puncta) and autolysosomes (red LC-3 puncta) per cell. **(D)** Cells treated with docetaxel (2 nM) for different times with or without bafilomycin (10 μM) (upper panel) and cells treated with various concentrations of docetaxel with or without bafilomycin (10 μM) for 4 8h (lower panel). Expression of autophagic markers LC-3I/II, BECN1, and ATG5 was detected by Western blotting. **(E)** Expression of autophagic markers in cells treated with rapamycin or cultured in a starving condition. **(F)** Cells were treated with different concentrations of docetaxel for 48 h or with 2nM docetaxel for different times, and levels of p-AMPK, p-mTOR, and p-P70S6K were detected by Western blotting. **(G)** KLF5 expression and autophagic marker change in cells after treatment with P70S6K inhibitor (6μM) and docetaxel (2 nM) together or separately were detected by Western blotting. *p<0.05, ** p<0.01.

Although low KLF5 expression enhanced cell resistance to docetaxel, the relationship between KLF5 expression and docetaxel treatment remained unclear. Thus, we determined the KLF5 protein level with docetaxel treatment at various concentrations or at varying time points with a concentration of 2 nM and observed downregulation of KLF5 as displayed in Figures 2A and 2B. Previous studies showed that autophagy affected cell toxicity of docetaxel. We employed a pH-sensitive LC-3 construct consisting of a tandem fusion of the acid-insensitive mRFP and the acid-sensitive EGFP in cells treated with docetaxel. Consistently, a pronounced increase in both yellow and red puncta was observed in C4-2 and CW22RV1 cells treated with docetaxel (Figure 2C). Bafilomycin A1 (Baf1), an autophagy inhibitor that can affect the acidification of lysosomes rendering them nonfunctional to digest LC-3 II and other contents, was used. As expected, docetaxel treatment increased LC-3 II accumulation and protein expression of BECN1 and ATG5, with or without Baf1, in both time- and dose-dependent manner in C4-2 and CW22RV1 cells (Figure 2D) which suggested that docetaxel affected autophagy by modulating the steps upstream of lysosome function. Taken together, these results indicated that docetaxel suppresses KLF5 expression and induces cell autophagy in prostate cancer cells.

### Docetaxel suppresses KLF5 expression via AMPK/mTOR/p70S6K signaling

To further identify and confirm the expression of KLF5 under autophagy condition, rapamycin treatment and starvation, the classic triggers of autophagy, were used. As shown in Figure 2E, autophagy was successfully induced, and KLF5 expression was significantly suppressed by rapamycin treatment and starvation. It has been reported that docetaxel treatment can induce the phosphorylation of AMPK, which plays an important role in the induction of cell autophagy. We, therefore, hypothesized that docetaxel treatment downregulated KLF5 via AMPK/mTOR pathway. To verify this hypothesis, we examined the activity of AMPK and downstream effectors in C4-2 and CW22RV1 cells and found that p-AMPK level was increased while p-mTOR and p-p70S6K (ribosomal protein S6 kinase) levels and KLF5 expression were decreased by docetaxel treatment (Figure 2F). Also, to detect whether p-p70S6K promotes KLF5 expression, we treated C4-2 and CW22RV1 cells with PF4708671, an inhibitor of p70S6K, and found increased LC3-II accumulation and BECN1 expression but decreased p62 and KLF5 levels (Figure 2G). These results suggested that docetaxel suppresses KLF5 expression via AMPK/mTOR/p70S6K signaling.

### Docetaxel-induced cell autophagy is impaired by KLF5 knockdown in prostate cancer cells

**Figure 3:**
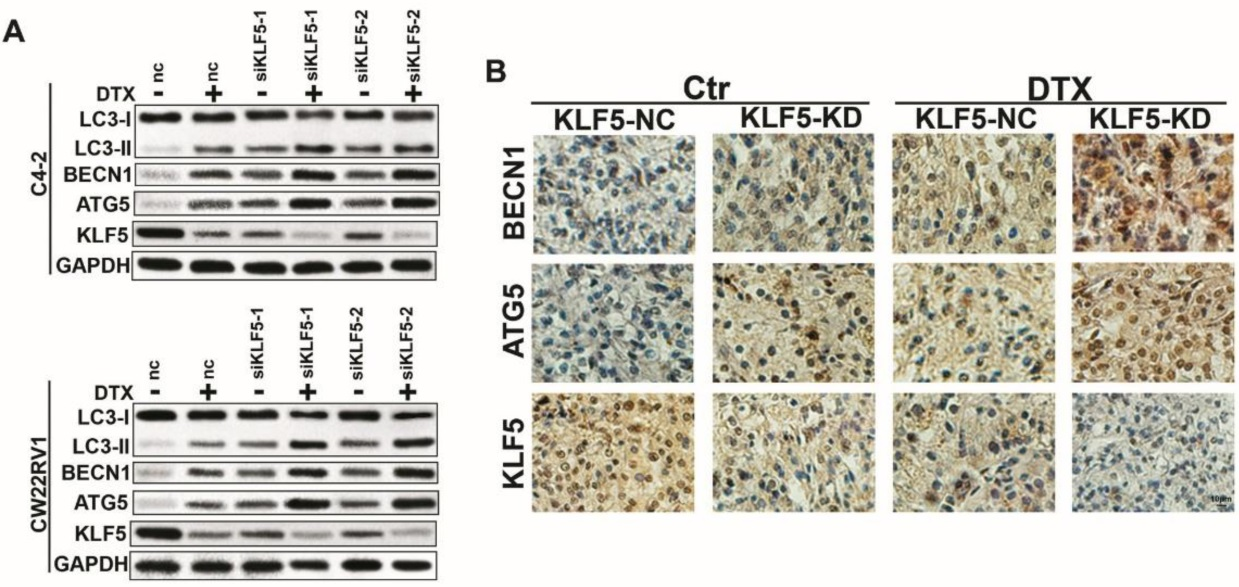
** Cell autophagy is impaired after KLF5 knockdown in prostate cancer cells. (A)** Cells transfected with siKLF5 or a scrambled RNA for 24 h and then treated with docetaxel (2 μM) for 48 h. Autophagic markers LC-3I/II, BECN1, and ATG5 were detected by Western blotting. **(B)** Representative photographs (400×) of immunohistochemistry (IHC) staining of KLF5, ATG5, and KLF5 expression in paraffin-fixed tumor sections from docetaxel-treated CW22RV1/shNC and CW22RV1/shKLF5 xenografts. Bars = 20 μm.

Since docetaxel downregulated KLF5 expression and induced cell autophagy simultaneously, we further investigated whether KLF5 inhibited cell autophagy induced by docetaxel treatment. As shown in Figure 3A, the knockdown of KLF5 amplified the increase in LC3-II accumulation, BECN1 expression, and ATG5 expression by docetaxel treatment. Consistent with this observation in vitro, in CW22RV1 xenograft tumors, KLF5 knockdown group with docetaxel treatment showed the highest BECN1 and ATG5 protein levels as detected by immunohistochemistry (Figure 3B). These results suggested that KLF5 knockdown increases cell autophagy induced by docetaxel treatment in prostate cancer cells.

### KLF5 knockdown increases cell autophagy in C4-2 and CW22RV1 cells

**Figure 4:**
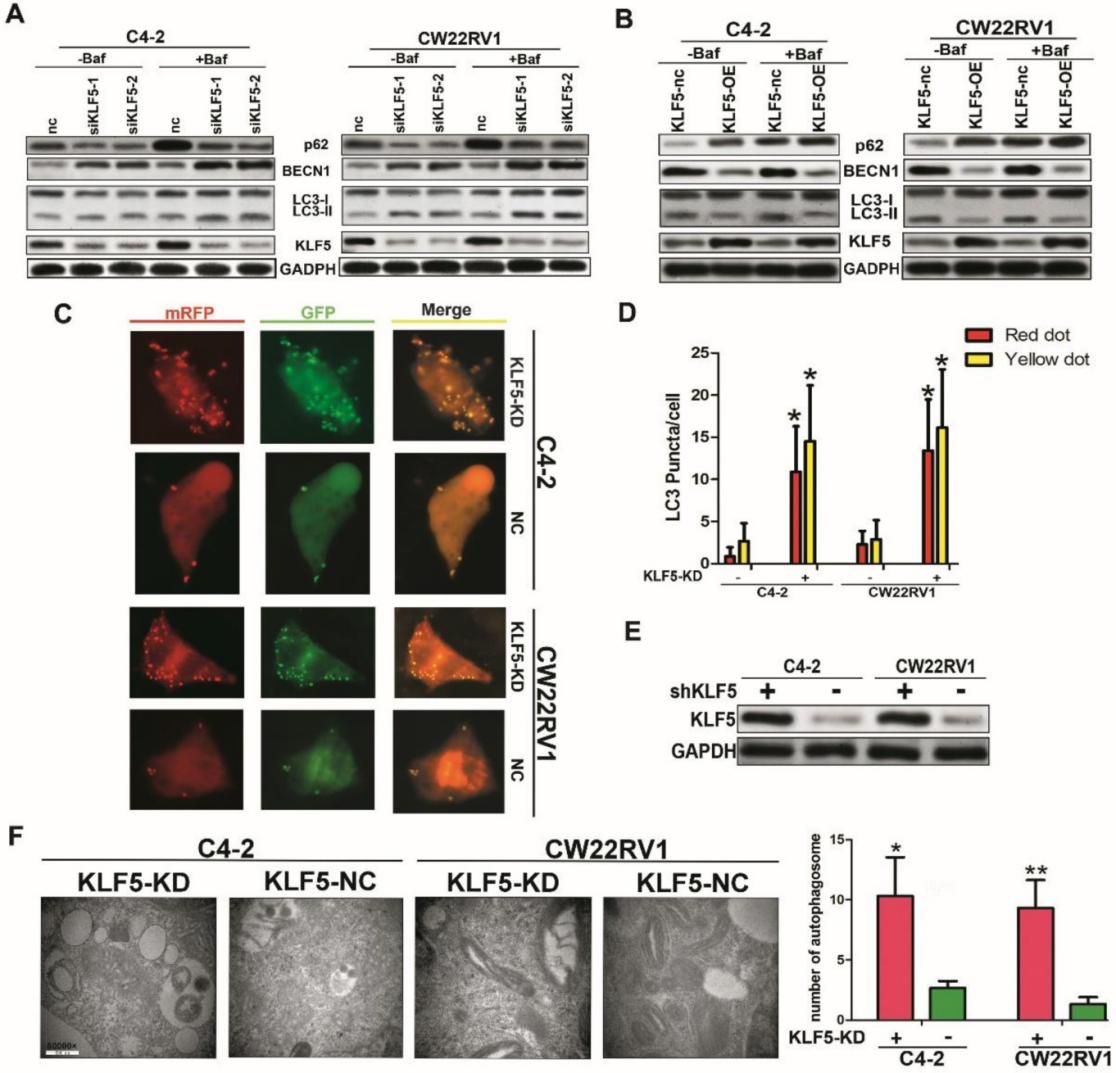
** KLF5 knockdown increases cell autophagy in C4-2 and CW22RV1 cells. (A)** Cells were transfected with siKLF5 or a scrambled RNA for 48 h, and autophagic markers LC3I/II, BECN 1, and p62 were analyzed by western blotting. **(B)** Autophagic markers were detected in cells with overexpressed KLF5 or empty vector (KLF5-nc). **(C)** Examples of cells transiently transfected with ptfLC-3 plasmid and transfected with siKLF5 for 48 h by fluorescence microscopy (×400). Yellow and red dots point to autophagosomes and autolysosomes, respectively. **(D)** Quantification of the number of autophagosomes (yellow LC-3 puncta) and autolysosomes (red LC-3 puncta) per cell. (E) Knockdown of KLF5 was validated by Western blotting (upper panel). **(F)** Representative figures of cell ultrastructure observed by TEM and quantification of the number of autophagic double-membrane compartments. Right: the quantitative data. *p<0.05, ** p<0.01.

To further explore the role of KLF5 in cell autophagy, we applied specific siRNA to knockdown KLF5 in C4-2 and CW22RV1 prostate cancer cell lines and examined the expression of autophagy-related markers using Western blotting. As displayed in Figure 4B, compared to the control, KLF5 downregulation significantly increased LC-3 II accumulation and BECN1 expression and decreased p62 level, the substrate of autophagy. Conversely, overexpressed KLF5 resulted in less LC3-II accumulation, lower BECN1 level, and higher p62 level (Figure 4C). We next transiently transfected a pH-sensitive LC-3 construct consisting of a tandem fusion of the acid-insensitive mRFP and the acid-sensitive EGFP into prostate cancer cells to monitor the effects of KLF5 knockdown on the formation of autophagosome and its matured form of autolysosome. A pronounced increase in both yellow and red puncta was observed in C4-2 and CW22RV1 cells with KLF5-knockdown (Figure 4D-4E). These results implied that KLF5-knockdown enhances autophagosome formation and maturation in prostate cancer cells.

The autophagy induction was further confirmed by TEM to observe the ultrastructure of the cells. KLF5-knockdown significantly increased autophagic double-membrane compartments containing lamellar structures (Figure 4F). In summary, these results indicated that KLF5 plays an important role in suppressing cell autophagy in prostate cancer cells.

### KLF5 binds on the promoter of *BECN1* cooperatively with HDAC3 and suppresses the transcription of BECN1

**Figure 5:**
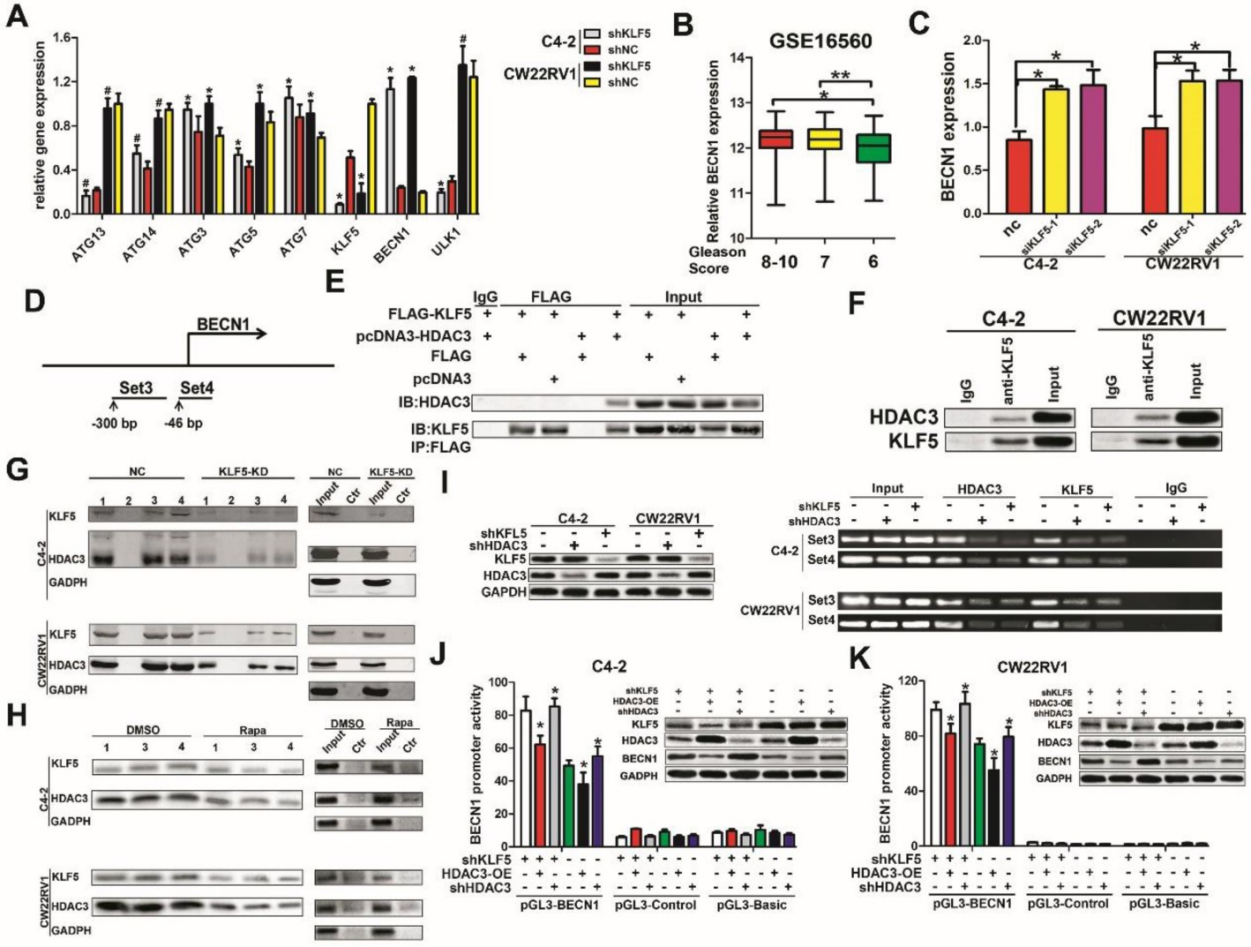
** KLF5 interacts with HDAC3 and binds together on the promoter of *BECN1* to inhibit transcription. (A)** Knockdown of KLF5 elevated the expression of BECN1 but not other important autophagy-related genes, as detected by RT-qPCR. **(B)** Relative expression of BECN1 in prostate cancer patients with different Gleason Scores. Gleason Score 6:n=83; Gleason Score 7:n=117; Gleason Score 8-10:n=81. Date from GEO database (GSE 16560). **(C)** C4-2 and CW22RV1 cells were transfected with indicated siRNAs to knockdown KLF5. The relative BECN1 expression was detected by RT-qPCR and normalized to GAPDH expression. The results are shown as the mean ± SD. **(D)** Representation of *BECN1* promoter region as mapped by oligonucleotides pulldown assays and ChIP assay. **(E)** Co-immunoprecipitation of KLF5 and HDAC3 proteins in HEK293 cells. **(F)** Co-immunoprecipitation of endogenous KLF5 and HDAC3 proteins in C4-2 and CW22RV1 cells. **(G)** Knockdown of KLF5 decreased the binding of HDAC3 on *BECN1* promoter as detected by oligonucleotides pulldown assays. The “1, 2, 3, and 4” indicate four different oligonucleotides, which locate in the proximal region (-255 to +132 bp) of the *BECN1* promoter. **(H)** Rapamycin treatment decreased binding of KLF5 and HDAC3 on *BECN1* promoter as detected by oligonucleotide pulldown assays. Ctr: The DNA in TE buffer was used as a negative control to perform the oligonucleotide pulldown assay. **(I)** Binding of HDAC3 and KLF5 on the *BECN1* promoter in C4-2 and CW22RV1 cells as detected by the ChIP assay. The protein expression of KLF5, HDAC3, and BECN1 was detected by Western blotting.** (J and K)** Dual-fluorescence assay showed knockdown of KLF5 increased the inhibition of *BECN1* promoter activity by HDAC3 in C4-2 (J) and CW22RV1 (K) cells. #*p*>0.05, *p<0.05, ** p<0.01.

To investigate the underlying mechanism of suppression of cell autophagy by KLF5, we examined the effect of KLF5 on the expression of important autophagy-related genes, such as ATG3, ATG5, ATG7, ATG13, ATG14, BECN1, and ULK1. We found that when KLF5 was knocked down by shRNA, among all genes tested, only the expression of BECN1 was significantly increased in C4-2 and CW22RV1 cells (Figure 5A). This result was consistent with our previous observations of elevated expression of BECN1 when KLF5 was downregulated by docetaxel, rapamycin, and KLF5 siRNA (Figure 2A, 2D, 2E, 3A, and 4A). Since high BECN1 level was associated with high-grade prostate cancer (Figure 5B, GSE16560), we further investigated the regulation of BECN1 expression by KLF5. RT-qPCR results showed that KLF5 knockdown by siRNA significantly increased BECN1 mRNA level (Figure 5C). Also, treatment of KLF5 knockdown and control prostate cancer cells with MG132, a proteasome inhibitor, for different times did not show a significant difference in the BECN1 protein levels between the two groups (Figure S1A-S1B). These results suggested that KLF5 knockdown upregulates BECN1 expression through its transcription rather than protein stability.

Given that KLF5 is a transcription factor, we investigated whether it regulated the transcription of BECN1. It has been reported that KLF5 could interact with HDAC1, an enzyme that removes acetyl groups from histone, in the transcription complex 17. As HDAC3 is a member of histone deacetylases and also involved in the transcription of BECN1 18, we speculated that KLF5 might work in concert with HDAC3 to regulate BECN1 transcription. To test this hypothesis, *BECN1* promoter regions containing GC boxes with high binding affinity to KLF5 were selected and amplified with biotinylated primers (Figure 5D). First, we examined the interaction between KLF5 and HDAC3. By employing co-immunoprecipitation assay, we observed that exogenous KLF5 interacted with HDAC3 in HEK293 cells (Figure 5E) and endogenous KLF5 interacted with HDAC3 in C4-2 and CW22RV1 cells (Figure 5F). Furthermore, as shown in Figure 5G, both KLF5 and HDAC3 could bind to the promoter of *BECN1*, while KLF5 knockdown attenuated the HDAC3 binding as detected by oligonucleotides pulldown assay and Western blot analysis. To detect the binding of KLF5 and HDAC3 on the *BECN1* promoter in cell autophagy, we treated C4-2 and CW22RV1 cells with rapamycin and performed oligonucleotides pulldown assay, and found that rapamycin decreased the binding of both KLF5 and HDAC3 on the promoter of *BECN1* (Figure 5H). To further verify whether KLF5 and HDAC3 bound to the promoter of *BECN1*, we applied ChIP assay and found that both KLF5 and HDAC3 bind to the promoter of *BECN1* and KLF5 knockdown decreased the binding of HDAC3 (Figure 5I). Collectively, these results implied that both KLF5 and HDAC3 bind to the promoter of *BECN1,* and the binding of HDAC3 is dependent on KLF5.

To detect whether the binding of KLF5 and HDAC3 to the *BECN1* promoter inhibited its transcription, we cloned the DNA fragment of *BECN1* promoter and inserted it into the pGL3-basic luciferase reporter plasmid and performed dual luciferase activity assays in C4-2, CW22RV1, and 293T cells. Compared to the control group, the knockdown of KLF5 increased, but overexpression of HDAC3 decreased *BECN1* promoter activity in both C4-2 and CW22RV1 cells (Figure 5J, 5K). On the other hand, KLF5 overexpression alone could not decrease the luciferase activity of *BECN1* promoter, but HDAC3 overexpression decreased *BECN1* promoter activity, and combined HDAC3 and KLF5 overexpression further decreased *BECN1* promoter activity in 293T cells (Figure S1C). Collectively, these results indicated that cooperative binding of KLF5 with HDAC3 on the promoter of *BECN1* inhibits the promoter activity and suppresses the transcription of BECN1.

The inhibitory effect of Bcl-2 on BECN1 and autophagy has been identified in many reports 19-21. Interestingly, we found that KLF5 knockdown significantly decreased Bcl-2 mRNA (Figure S2A) and protein levels (Figure S2B). Given these results, we examined the association of KLF5 and Bcl-2 mRNA expression in prostate cancer tissues in the TCGA database. As shown in Figure S2C, there was a correlation between the expression of Bcl-2 mRNA and KLF5 (R=0.296), which was consistent with our results. Furthermore, Bcl-2 knockdown in C4-2 and CW22RV1 cells upregulated autophagic markers (ATG5, ATG7, ATG3, and BECN1) and accumulated LC-3 II protein as detected by Western blot analysis. The increased LC-3 II levels in bafilomycin A1-treated cells indicated autophagy induction (Figure S2D). To better understand how KLF5 regulated Bcl-2 expression, we designed the primers amplifying the GC-rich regions and performed ChIP assay. The schematic binding map displayed in Figure S3E indicates that KLF5 bound to the Bcl-2 promoter in C4-2 and CW22RV1 cells. These results suggested that knocking down KLF5 transcriptionally promotes BECN1 and inhibits Bcl-2 expression. The downregulated Bcl-2 attenuates the inhibition of autophagy and might indirectly increase it.

### BECN1 is critical for the inhibition of cell autophagy by KLF5

**Figure 6:**
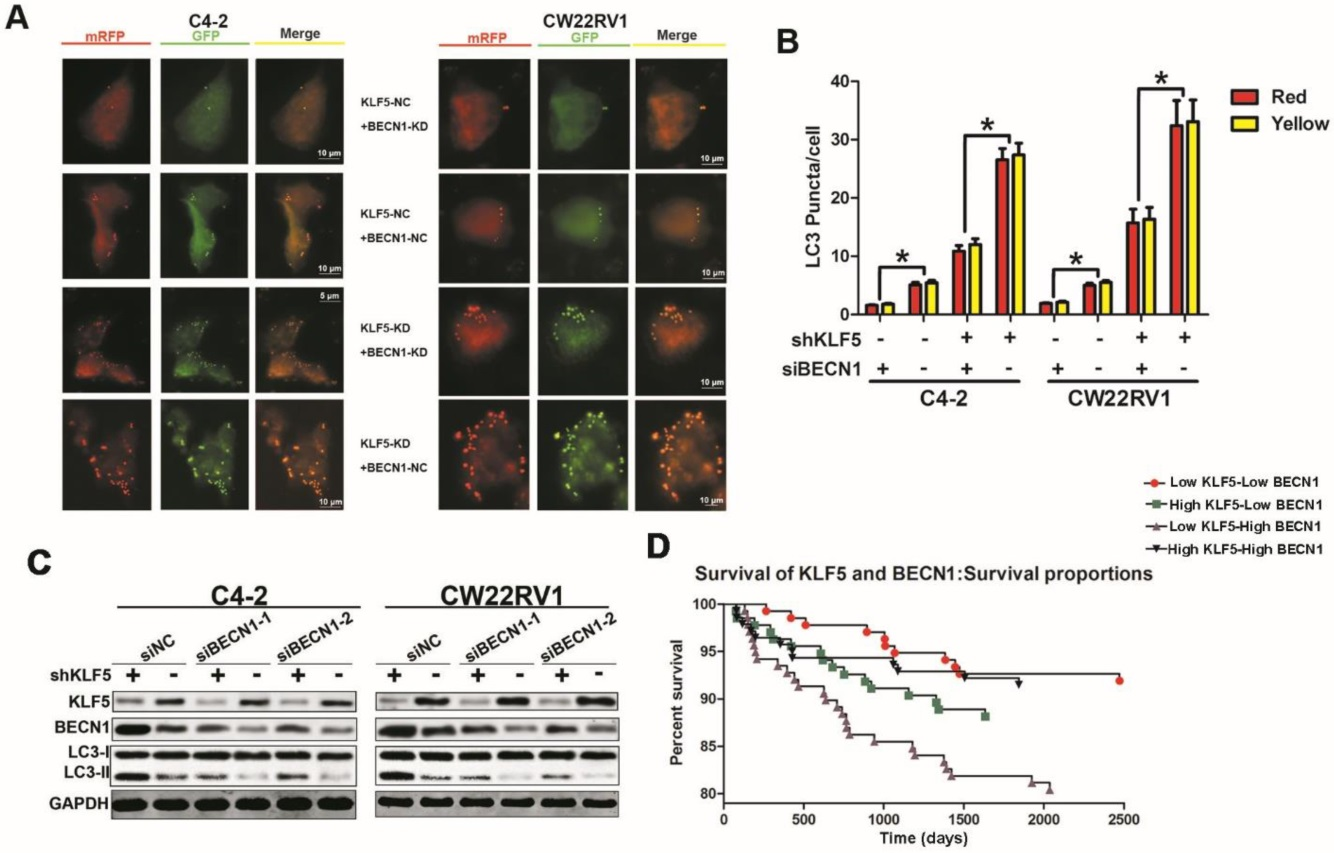
** BECN1 is critical for KLF5 to suppress cell autophagy. (A)** Examples of shKLF5-cells transiently transfected with ptfLC-3 plasmid and transfected with siBECN1 for 48 h by fluorescence microscopy (×400). Yellow and red dots point to autophagosomes and autolysosomes, respectively. **(B)** Quantification of the number of autophagosomes (yellow LC-3 puncta) and autolysosomes (red LC-3 puncta) per cell. *p<0.05.** (C)** Cells with KLF5 knockdown by shRNA transfected with siBeclin1 or a scrambled RNA. Autophagic markers LC-3I/II and BECN1 were detected by Western blotting. **(D)** Kaplan-Meier analysis of the first recurrence of prostate cancer patients with low KLF5low BECN1 expression (n=136), high KLF5-low BECN1 expression (n=135), low KLF5-high BECN1 expression (n=138), and high KLF5-high BECN1 expression (n=140). *P* = 0.0026 between the groups with low KLF5 expression-low BECN1 expression and the group with low KLF5 expression-high BECN1 expression; *P* = 0.0195 between the group with low KLF5-expression high BECN1 expression and the group with high KLF5 expression-low BECN1 expression. Data from the TCGA database.

Our results revealed that KLF5 suppressed BECN1 transcription, and the BECN1 protein has been shown to play a critical role in regulating cell autophagy 22. We further employed the pH-sensitive LC-3 construct consisting of a tandem fusion of the acid-insensitive mRFP and the acid-sensitive EGFP in cells with KLF5 knockdown or knockdown of both KLF5 and BECN1. KLF5 knockdown significantly enhanced red and yellow puncta, while BECN1 knockdown impaired this increase of puncta (Figure 6A-6B). Also, BECN1 knockdown reduced the promotion of LC3-II accumulation induced by KLF5 knockdown in both C4-2 and CW22RV1 cells, as detected by Western blotting (Figure 6C). These results indicated that the induction of BECN1 expression was critical for KLF5 knockdown to increase cell autophagy. We further analyzed the prognosis (recurrence time) of prostate cancer patients from the TCGA database and divided the patients into four cohorts according to the expression levels of KLF5 and BECN1 (Figure 6D). Prognosis of low KLF5 /high BECN1 expression cohort was the worst compared with the three other cohorts (p=0.0099) which suggested that the level of KLF5/BECN1 axis was significantly associated with recurrence of prostate cancer.

Since Bcl-2 plays a role in autophagy, we further analyzed the TCGA database of prostate cancer patients and found the highest percentage of high-grade tumors in the cohort with low KLF5/high Bcl-2 levels. (Gleason Score greater than 8). However, Bcl-2 expression was not significantly correlated with the Gleason Score or Pathological N of prostate cancer (Figure S3D-3E), and overall survival of prostate cancer patients (Figure S3C). These results indicated that the regulatory role of KLF5/BECN1 regulation rather than that of KLF5/Bcl-2 is related to the recurrence of prostate cancer.

### Repression of BECN1 and cell autophagy is necessary for KLF5 to increase cell sensitivity to docetaxel

**Figure 7:**
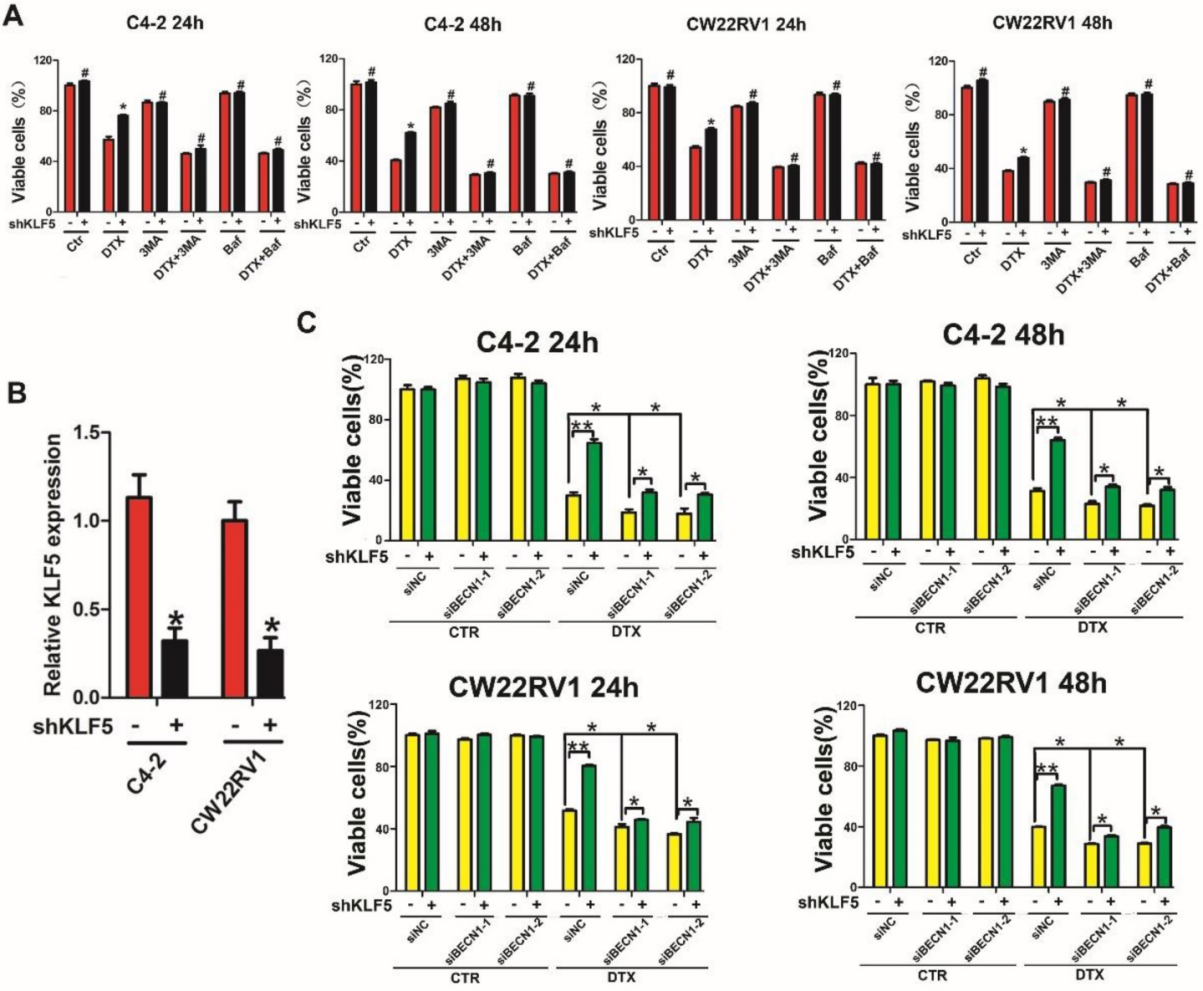
** KLF5 regulates docetaxel sensitivity by modulating cell autophagy. (A)** Autophagy inhibitors diminished the effect of KLF5 knockdown on the docetaxel sensitivity of C4-2 and CW22RV1 cells.** (B)** KLF5 knockdown was validated by RT-qPCR analysis.** (C)** Cells with KLF5 knockdown by shRNA transfected with siBECN1 or a scrambled RNA and treated with docetaxel (2 nM) for different times. Cell viability was assessed by the MTT assay. #*p*>0.05, *p<0.05, ** p<0.01.

Since KLF5 knockdown augmented cell resistance to docetaxel as well as increased BECN1 expression and cell autophagy induced by docetaxel, we investigated whether KLF5 increased cell sensitivity to docetaxel by inhibiting BECN1 and cell autophagy. MTT assay showed increased cell death with autophagy inhibition (treatment with 3MA and Baf1, which are pharmacological inhibitors of autophagy) and enhanced cell viability with KLF5 knockdown when treated with docetaxel. These observations suggested that autophagy positively contributed to cell survival with docetaxel treatment and autophagy inhibition impaired the diminished cell sensitivity to docetaxel caused by KLF5 knockdown (Figure 7A). Furthermore, we found that, compared with the control group, KLF5 knockdown increased cell viability when treated with docetaxel; however, this increase was reduced by BECN1 knockdown (Figure 7B), indicating that repression of BECN1 was necessary for KLF5 to regulate cell sensitivity to docetaxel. It is of note that partially blocking BECN1, but not totally impairing the KLF5-knockdown induced increased cell sensitivity to docetaxel, which indicated that KLF5 suppresses autophagy only partially by depressing BECN1.

Taken together, our results indicated that KLF5 knockdown enhances docetaxel-induced cell autophagy, and repression of BECN1 and cell autophagy is necessary for KLF5 to increase cell sensitivity to docetaxel in prostate cancer cells.

## Discussion

**Figure 8:**
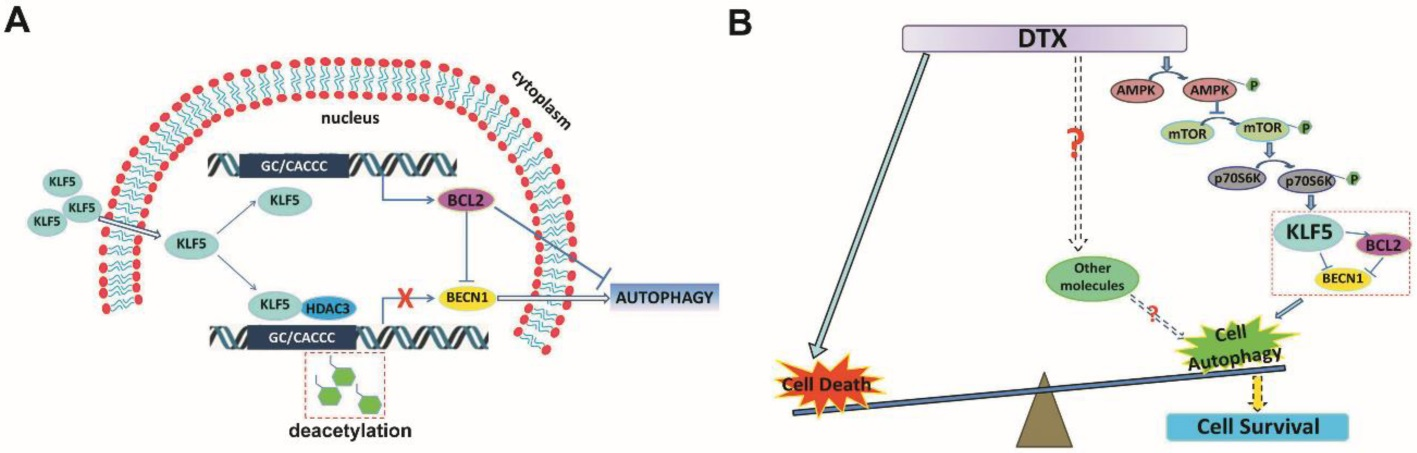
** Schematic presentation of the mechanisms. (A)** A schematic presentation of the mechanism of KLF5 regulation of BECN1 expression. **(B)** A schematic presentation of the mechanism by which KLF5 down-regulation promotes docetaxel-induced cell autophagy and protects cell death in prostate cancer.

KLF5 is frequently deleted and down-regulated in prostate cancer. We found that KLF5 down-regulation was associated with progression of prostate cancer and poor prognosis of patients. KLF5 knockdown reduced the sensitivity of prostate cancer cells to docetaxel *in vitro* and *in vivo*, and docetaxel treatment decreased the expression of KLF5. It has been reported that inhibition of autophagy could dramatically increase the sensitivity of cells to docetaxel treatment in prostate cancer 10. Herein we confirmed that docetaxel treatment induced autophagy in prostate cancer cells which provided protection from cell death. Thus, we hypothesized that KLF5 could be a regulator of cell autophagy. Interestingly, we found that KLF5 could inhibit prostate cancer cell autophagy via suppressing the transcription of BECN1 cooperatively with HDAC3 (Figure 8A). Furthermore, BECN1 was critical for KLF5 to inhibit cell autophagy, and repression of BECN1 and cell autophagy was necessary for KLF5 to increase cell sensitivity to docetaxel. In prostate cancer, docetaxel treatment repressed KLF5 expression via AMPK/mTOR/p70S6K signaling pathway, which may lead to increased BECN1 expression, induction of cell autophagy and promotion of cell survival (Figure 8B). Thus, our results indicated that down-regulation of KLF5 in castration-resistant prostate cancer might not only promote cell autophagy but also facilitate cell autophagy induced by docetaxel, leading to increased cell survival and reduced cell sensitivity to docetaxel.

It has been reported that chromosome 13q21 deletion in human primary and metastatic prostate cancer loci 23 led to the downregulation of KLF5. We found that KLF5 downregulation may result in low sensitivity to docetaxel in prostate cancer cells. On the contrary, KLF5 overexpression increased the sensitivity of prostate cancer cells to docetaxel (Figure S4A, S4C), suggesting that expression level of KLF5 in prostate cancer might be indicative of the cancer response to docetaxel treatment; the threshold of the KLF5 level remains to be identified though. This information might be helpful in identifying whether prostate cancer patients would respond well to docetaxel. Also, not only the low expression of KLF5 but the low level of KLF5/high level of BECN1 are associated with the poor prognosis of prostate cancer patients and have a potential value in precision medicine for the prognostic evaluation of patients.

Currently, docetaxel is a standard-of-care in men with symptomatic CRPC and can extend patient survival for approximately 2 months 3. The reasons for this limited therapeutic efficacy and why some patients do not respond to docetaxel treatment are not known. Some researchers have linked taxanes with autophagy, and it was reported that the taxane paclitaxel could induce autophagy in tumor cells 24, 25, though the mechanism was not clear. Besides the known direct phosphorylation of ULK-1 or BECN1 by AMPK 26, 27, in the present study, we found that docetaxel treatment enhanced the AMPK activity and increased cell autophagy through a novel mTOR/p70S6K/KLF5/BECN1 signaling pathway. P70S6K is a key downstream effector of mTOR, which plays a crucial role in the regulation of protein biosynthesis, autophagy, cell growth, and cell proliferation 28, 29. mTORC1 directly phosphorylates S6K1, which can promote mRNA translation initiation through activating eIF4B or enhancing the degradation of PDCD4, an inhibitor of eIF4B 29-31. We found that expression of KLF5 protein was down-regulated by the p70S6K inhibitor (Figure 2G), suggesting that p70S6K could promote KLF5 expression by enhancing initiation of its mRNA translation in prostate cancer cells and KLF5 could mediate p70S6K function in autophagy.

Since autophagy is believed to protect tumor cells from stress-induced cell death 28, 29, 32, 33, pharmacological inhibitors of autophagy were tested in clinical trials for their possible role in sensitizing malignant cells to therapy. However, autophagy is important for the proliferation, survival, and effector functions of various immune cells responsible for tumor inhibition, and autophagy inhibitors would eliminate these cells 34, 35. Also, inhibition of autophagy may promote the malignant transformation of cells in the healthy tissues 36. Therefore, identifying and targeting the specific molecular alterations that promote autophagy in tumor cells but not in normal cells may be a safe way to antagonize cell autophagy induced by chemotherapy. In the present study, our findings suggest that protection of KLF5 protein level, for example, through suppression of E3 ligases targeting KLF5, may more specifically impede the docetaxel-induced autophagy. Since data on the relationship between gene expression levels and tumor sensitivity to docetaxel is non-existent or scarce, it is difficult to analyze whether KLF5 expression level is associated with tumor response to docetaxel in prostate cancer patients. Therefore, such specific approaches need to be further investigated in the future.

## Conclusions

Our results indicate that in prostate cancer, docetaxel treatment represses KLF5 expression through AMPK/mTOR/p70S6K signaling pathway, which may lead to increase of BECN1, induction of cell autophagy, and promotion of cell survival. Thus, KLF5 appears to be an important repressor of cell autophagy through regulation of BECN1. The correlation between KLF5/BECN1 levels and prostate cancer prognosis may be helpful toward precision medicine in the clinic by identifying prostate cancer patients, who are more likely to respond to docetaxel treatment.

## Supplementary Material

Supplementary figures and tables.

## Abbreviations

BECN1: beclin 1
Baf1: bafilomycin A1
CRPC: castration-resistant prostate cancer
GEO: Gene Expression Omnibus
KLF5: kruppel-like factor 5
TCGA: The Cancer Genome Atlas
TEM: transmission electron microscopy
