# KLF5 downregulation desensitizes castration-resistant prostate cancer cells to docetaxel by increasing BECN1 expression and inducing cell autophagy: Erratum

**DOI:** 10.7150/thno.85499

**Published:** 2023-05-15

**Authors:** Jing Jia, Hai-Bao Zhang, Qi Shi, Chao Yang, Jian-Bin Ma, Bin Jin, Xinyang Wang, Dalin He, Peng Guo

**Affiliations:** 1 Department of Urology, The First Affiliated Hospital of Xi'an Jiaotong University, Xi'an, Shaanxi, China, 710061; 2 Department of Plastic, Cosmetic and Maxillofacial Surgery, The First Affiliated Hospital of Xi'an Jiaotong University, Xi'an, Shaanxi, China, 710061; 3 Key Laboratory for Tumor Precision Medicine of Shaanxi Province, Xi'an, Shaanxi, China, 710061; 4 Oncology Research Lab, Key Laboratory of Environment and Genes Related to Diseases, Ministry of Education, Xi'an, Shaanxi, China, 710061

The authors regret that the original version of our paper, unfortunately, contained incorrect pictures in Figure 2, where the GADPH bands were mistakenly used for similar experiments. The correct version of Figure 2 is shown below.

The correction made in this erratum does not affect the original data and conclusions. The authors apologize for any inconvenience that the errors may have caused.

## Figures and Tables

**Figure 2:**
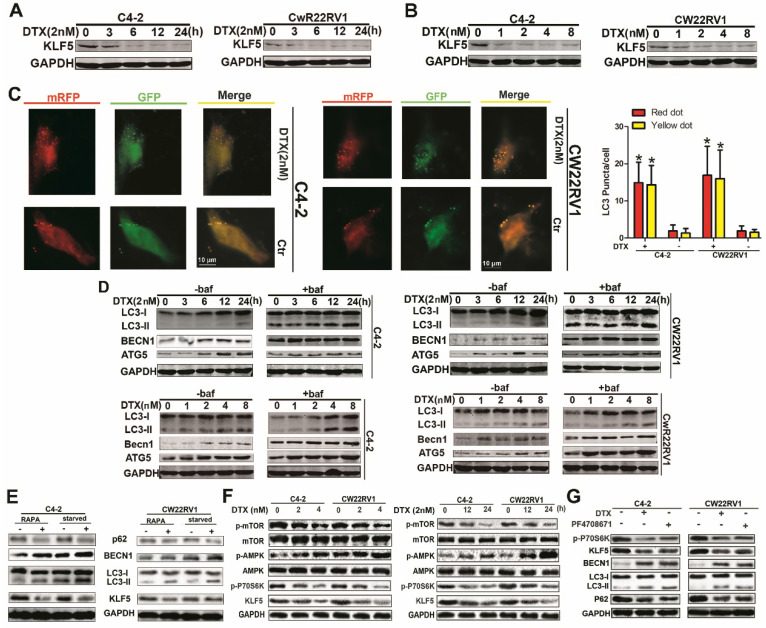
** Docetaxel reduces expression of KLF5 and increases cell autophagy in C4-2 and CW22RV1 cells. (A and B)** Cells treated with various concentrations of docetaxel for 4 8h or treated with docetaxel (2 nM) for different times. KLF5 protein level was detected by Western blotting. **(C)** Examples of cells treated with docetaxel for 24 h and then transiently transfected with ptfLC-3 plasmid for another 24 h by fluorescence microscopy (×400). Yellow dots point to autophagosomes while red dots point to autolysosomes. Right: Quantification of the number of autophagosomes (yellow LC-3 puncta) and autolysosomes (red LC-3 puncta) per cell. **(D)** Cells treated with docetaxel (2 nM) for different times with or without bafilomycin (10 μM) (upper panel) and cells treated with various concentrations of docetaxel with or without bafilomycin (10 μM) for 4 8h (lower panel). Expression of autophagic markers LC-3I/II, BECN1, and ATG5 was detected by Western blotting. **(E)** Expression of autophagic markers in cells treated with rapamycin or cultured in a starving condition. **(F)** Cells were treated with different concentrations of docetaxel for 48 h or with 2nM docetaxel for different times, and levels of p-AMPK, p-mTOR, and p-P70S6K were detected by Western blotting. **(G)** KLF5 expression and autophagic marker change in cells after treatment with P70S6K inhibitor (6μM) and docetaxel (2 nM) together or separately were detected by Western blotting. *p<0.05, ** p<0.01.

