# Supplementary material for: KLF5 downregulation desensitizes castration-resistant prostate cancer cells to docetaxel by increasing BECN1 expression and inducing cell autophagy
Source: Theranostics. 2019 Jul 28;9(19):5464–77. doi: 10.7150/thno.33282 (PMC6735397; doi:10.7150/thno.33282)
Supplement: Supplementary file 1 — Supplementary figures and tables. [file thnov09p5464s1.pdf]

Supplementary table 1

BCL2	R	CGGTTCAGGTACTCAGTCATCC
	F	GGTGGGGTCATGTGTGTGG
BECN1	R	GAATCTGCGAGAGACACCATC
	F	CCATGCAGGTGAGCTTCGT

Supplementary table 2. Primers for ChIP assay in *BECN1* promoter.

set1	F	5’-TGTTGGCCAGGCTGGTCTCG-3’
	R	5’-CTTCAGCGACTTCCCGGTAG-3’
set2	F	5’-CAGAGCGATGGTAGTTCTGG-3’
	R	5’-ATCCCAGCTACTCAGGAGGC-3’
set3	F	5’-ACTCCTGACCTCACGTGATC-3’
	R	5’-TACGACGGCAGCAGGAGCTC-3’
set4	F	5’-ACGTCCGGTCTCGGCGGAAG-3’
	R	5’-AAGTCCGGTCTACCGCGGAG-3’

Supplementary table 3. Oligonucleotides for pull down assay.

1	F	5’-biotin-CACCGCGCCCGCCGCCCCCTG-3’
	R	5’-biotin-CAGGGGGCGGCGGGCGCGGTG-3’
2	F	5’-biotin-AGCCCGGCCTCTGGGGGCCGCTGCCGGGCCT-3’
	R	5’-biotin-AGGCCCGGCAGCGGCCCCCAGAGGCCGGGC-3’
3	F	5’-biotin-TCGCTCCGGGGCCGACCCGA-3’
	R	5’-biotin-TCGGGTCGGCCCCGGAGCGA-3’
4	F	5’-biotin-AGTGCCTCCGCGGTAGACCGGA-3’
	R	5’-biotin-TCCGGTCTACCGCGGAGGCACT-3’

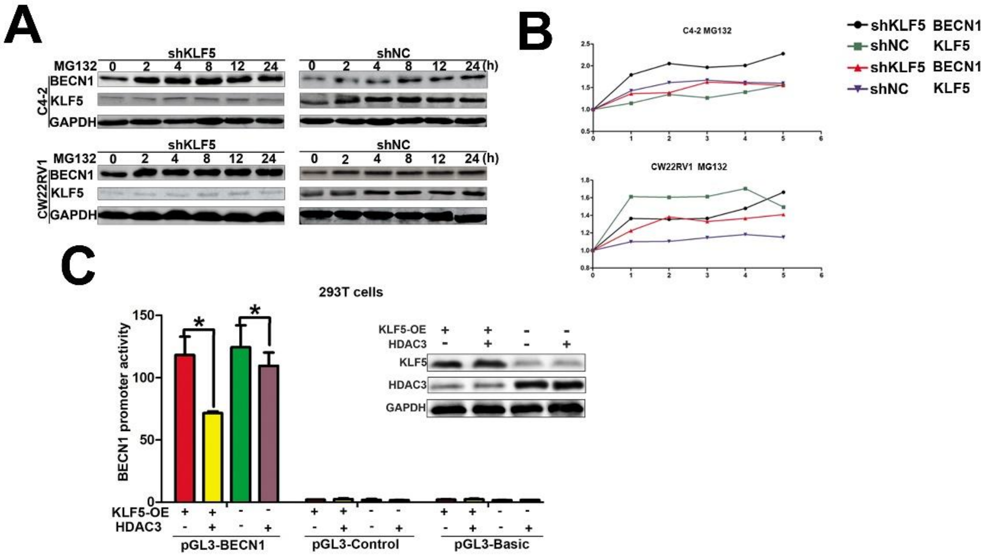

Figure S1. KLF5 upregulated BECN1 level not via affecting BECN1 protein stability and KLF5 cooperated with HDAC3 to repress *BECN1* promoter activity.

(A) Cells transfected with shKLF5 or a scrambled RNA and treated with MG132 for different times. Protein levels of BECN1 and KLF5 were detected by western blotting analysis. **(B)** Cells transfected with shKLF5 or a scrambled RNA and treated with MG132 for different times. Protein level of BECN1 and KLF5 were detected by western blotting analysis, and qualified and showed in curves. **(C)** Overexpressed HDAC3 inhibited *BECN1* promoter activity, and overexpressed both KLF5 and HDAC3 could further inhibit *BECN1* promoter activity as detected by dual-fluorescence assay in 293T cells.

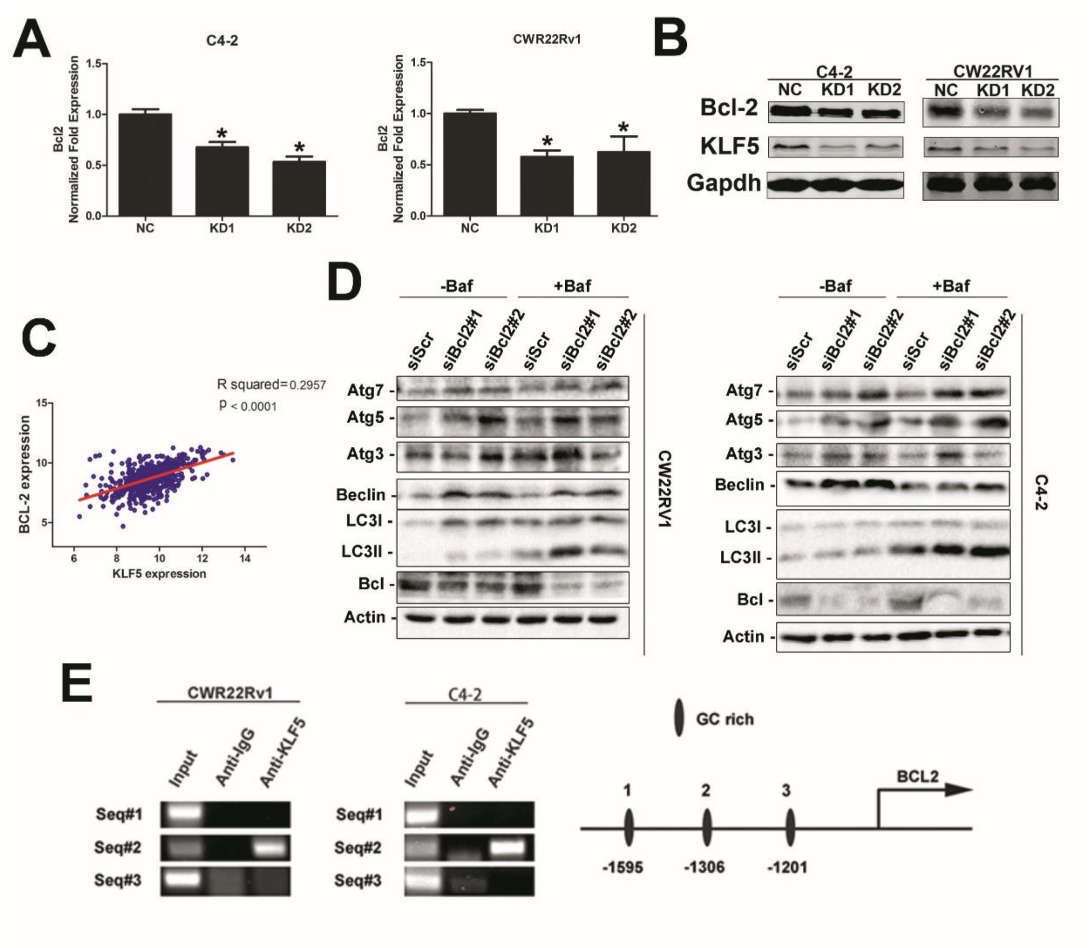

Figure S2. KLF5 upregulated Bcl2 through promoting its transcription.

(A and B) KLF5 knockdown by shRNA in cells and Bcl2 expression was detected by RT-qPCR and western blotting analysis. **(C)** Pearson's correlation analysis of KLF5 mRNA level and Bcl2 mRNA level in prostate cancer patients from TCGA database. **(D)** Cells transfected with siBcl2 or a scrambled RNA with or without Bafilomycin (10 μM). Autophagic markers LC-3I/II, BECN1, ATG3, ATG7 and ATG5 were detected by western blotting analysis. **(E)** Chromatin immunoprecipitation of KLF5 on the *Bcl2* gene promoter in C4-2 and CW22RV1 cells. Right: Representation of *Bcl2* promoter region as mapped by ChIP assay.

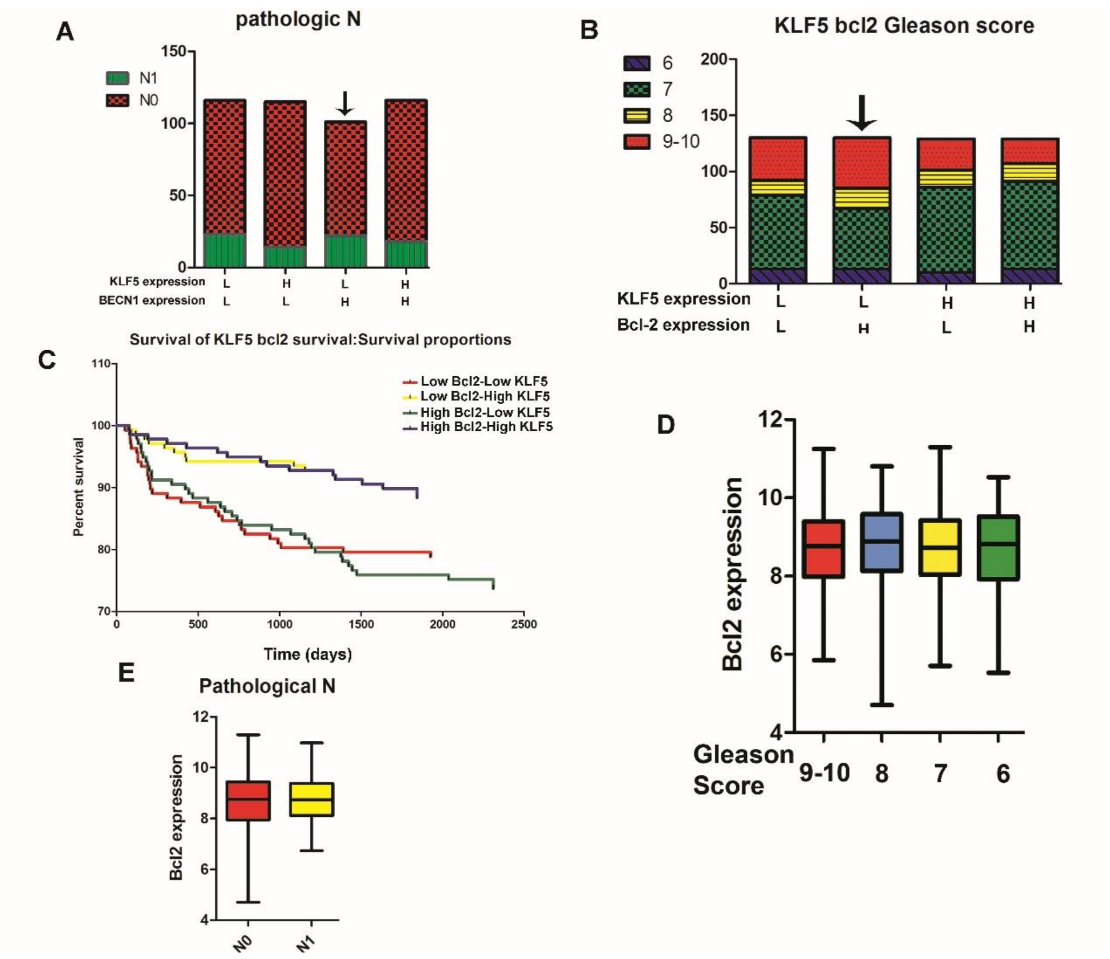

Figure S3. Bcl-2 expression was not associated with prostate cancer progression and prognosis of patients.

(A) Percentage of pathologic N1 in prostate cancer patients with low KLF5/low BECN1 expression (n=116), high KLF5/low BECN1 expression (n=115), low KLF5/high BECN1 expression (n=101) and high KLF5/high BECN1 expression (n=116).Data from TCGA database. **(B)** Percentage of various Gleason Score in prostate cancer patients with low KLF5/low Bcl2 expression (n=137), high KLF5/low Bcl2 expression (n=138), low KLF5/high Bcl2 expression (n=137) and high KLF5/high Bcl2 expression (n=138).Data from TCGA database. **(C)** Kaplan-Meier analysis of the first biochemical recurrence of prostate cancer patients with low KLF5/low Bcl2 expression (n=137), high KLF5/low Bcl2 expression (n=137), low KLF5/high Bcl2 expression (n=136) and high KLF5/high Bcl2 expression (n=137). *p*-value is depicted. Data from TCGA database. **(D)** Relative expression of Bcl2 in patients with different Gleason Scores. Gleason Score 6：n=49; Gleason Score 7：n=277; Gleason Score 8：n=63; Gleason Score 9-10：n=132. Date from TCGA database. **(E)** Relative expression of Bcl2 in patients with different Pathological N. N0: n=371, N1: n=71.

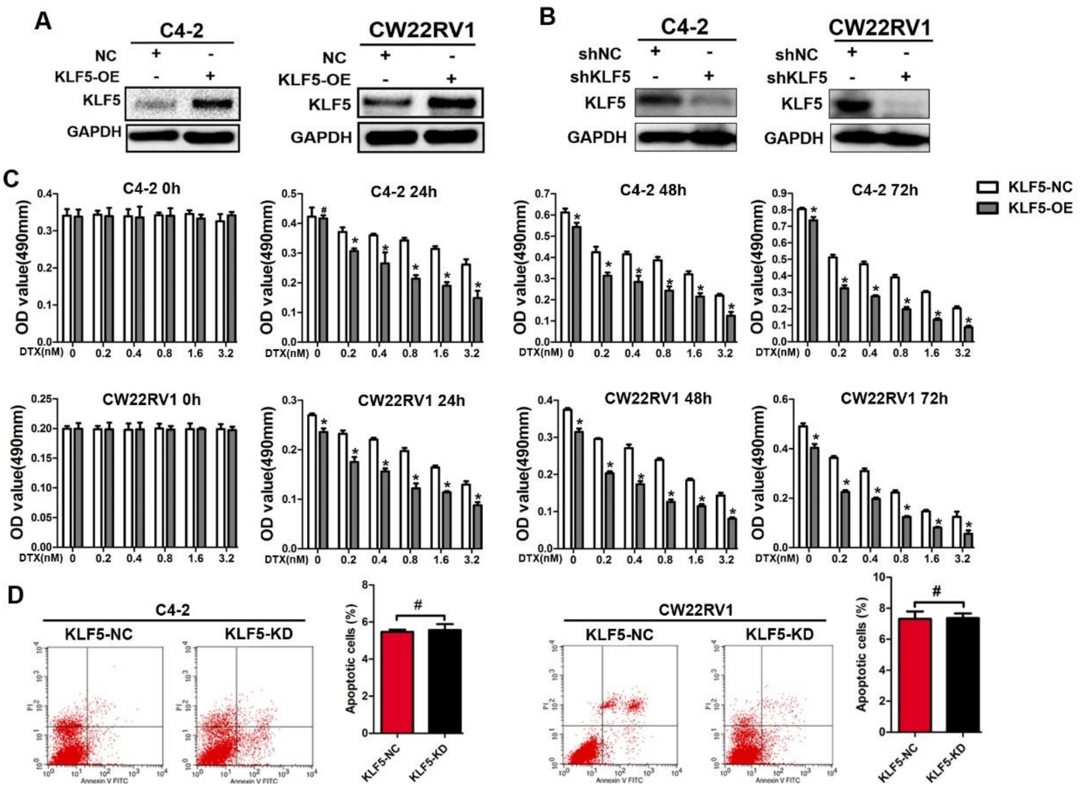

Figure S4. **(A, B)** Validation of KLF5 overexpression (A) or KLF5 knockdown (B) in C4-2 and CW22RV1 cells for the following assays. **(C)** Cells transfected with pcDNA3-KLF5 or empty vector for 48h and then treated with docetaxel of various concentration for different time. Cell viability was assessed by MTT assay. **(D)** Cell apoptosis was detected by FACS in KLF5 knockdown (KLF5-KD) and control cells. #p>0.05.
